# Family’s History Based on the *CDH1* Germline Variant (c.360delG) and a Suspected Hereditary Gastric Cancer Form

**DOI:** 10.3390/ijms21144904

**Published:** 2020-07-11

**Authors:** Laura Caggiari, Mara Fornasarig, Mariangela De Zorzi, Renato Cannizzaro, Agostino Steffan, Valli De Re

**Affiliations:** 1 Immunopathology and Cancer Biomarkers, Bioproteomic facility, Department of Translational Research, Centro di Riferimento Oncologico di Aviano (CRO), IRCCS, 33081 Aviano (PN), Italy; lcaggiari@cro.it (L.C.); mdezorzi@cro.it (M.D.Z.); asteffan@cro.it (A.S.); 2 Gastroenterology, Department of Medical Oncology, Centro di Riferimento Oncologico di Aviano (CRO), IRCCS, 33081 Aviano (PN), Italy; mfornasarig@cro.it (M.F.); rcannizzaro@cro.it (R.C.)

**Keywords:** *CDH1*, E-cadherin, HDGC, hereditary gastric cancer, genetic counseling

## Abstract

Hereditary diffuse gastric cancer (HDGC) is a cancer susceptibility syndrome caused by germline pathogenic variant in *CDH1*, the gene encoding E-cadherin. The germline loss-of-function variants are the only proven cause of the cancer syndrome HDGC, occurring in approximately 10–18% of cases and representing a helpful tool in genetic counseling. The current case reports the family history based on a *CDH1* gene variant, c.360delG, p.His121Thr in a suspected family for hereditary gastric cancer form. This frameshift deletion generates a premature stop codon at the amino acid 214, which leads to a truncated E-cadherin protein detecting it as a deleterious variant. The present study expands the mutational spectra of the family with the *CDH1* variant. Our results highlight the clinical impact of the reported *CDH1* variant running in gastric cancer families.

## 1. Introduction

The *CDH1* gene encodes the E-cadherin, a transmembrane calcium-dependent glycoprotein that mediates epithelial adhesions. Pathogenetic germline alterations in the *CDH1* are a cause of hereditary diffuse gastric cancer (HDGC) syndrome [1]. The identification of germline mutation carriers is a crucial step for the clinical management for the total gastrectomy recommendation. The cumulative risk for developing diffuse gastric cancer in *CDH1* mutation carriers is 70% by 80 years for men and 56 % for women [2], moreover, women carriers have an additional risk for lobular breast cancer and prophylactic mastectomy may be considered with family history [3].

The *CDH1* gene is located on chromosome 16 and consists of 16 exons [4]. E-cadherin is composed of five extracellular domains responsible for adhesive recognition, binding to the ectodomains of other cadherins presented on the neighboring cells forming epithelial integrity [5]. The E-cadherin receptor is linked to the fundamental intracellular processes through direct or indirect interaction between the E-cadherin cytoplasmic domain and a wide range of proteins [6].

The *CDH1* (c.360delG, p.His121Thrfs*94) variant was identified in a patient with gastric cancer (GC) [7]. We herein present the family pedigree of the brother of this patient (herein indicated as the proband) and the *CDH1* variant found in four of the six family members tested. The results support a hereditary form of GC associated with this *CDH1* variant. The deletion located in exon 3 results in a premature stop codon and leads to a non-functional protein.

## 2. Result

**Figure 1:**
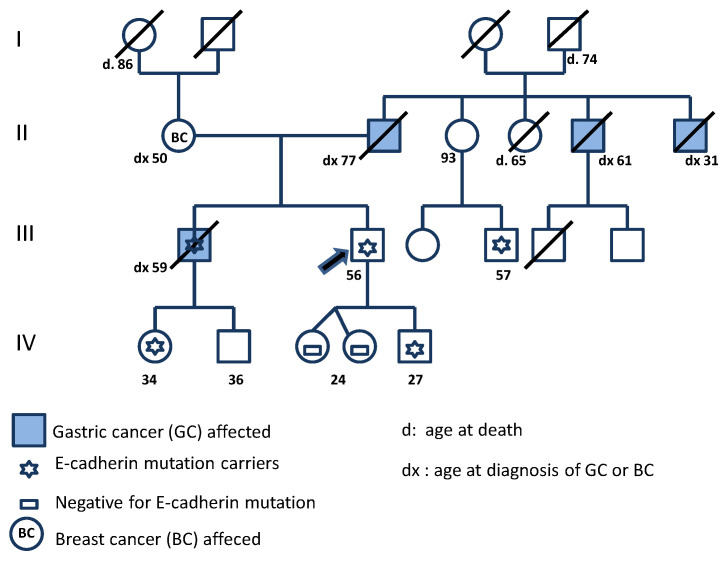
Family genetic pedigree with p.His 121Thrfs*94 variant in the *CDH1* gene. Arrow points to the proband. The shaded square and circle represent the male and female among the family members, respectively. Numbers indicate the age or the age at death in years; dx indicates the age at diagnosis, in years. Gastric cancer (GC); Breast cancer (BC).

The proband for this study is an asymptomatic 56 year-old Italian man. He had a family history of gastric cancer, including a brother who died from gastric cancer at the age of 59 years old (Figure 1). His father and two uncles were diagnosed to have gastric cancer and his mother died from breast cancer. *CDH1* germline molecularly testing was performed in the proband and in five family members: three sons, a niece, and a cousin.

**Figure 2:**
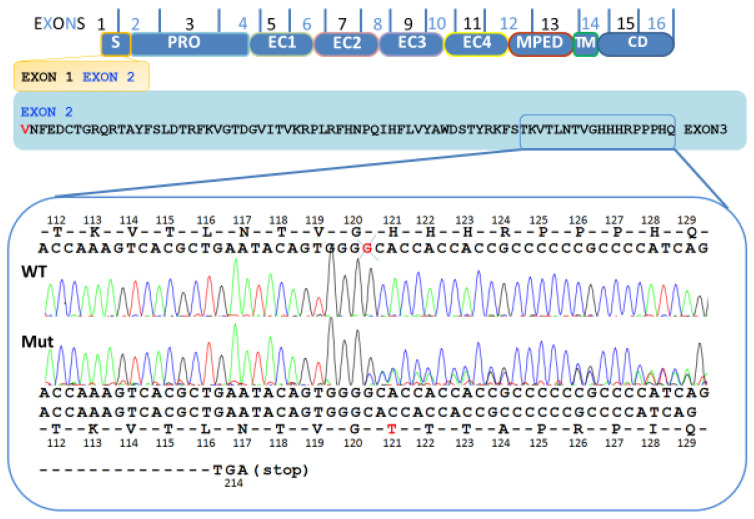
Schematic representation of the different domains of E-cadherin: signal peptide (S), precursor sequence (PRO), extracellular domains (EC1, EC2, EC3, EC4), membrane-proximal extracellular domain (MPED), transmembrane domain (TM) and a cytoplasmic domain (CD). Electropherogram of the *CDH1* variant gene in exon 3 (p.His121Thrfs*94), resulting in the new reading frame (94 codons up to and new stop codon (214)). Top: partial *CDH1* exon 3 wildtype (WT) sequence; bottom: the parallel *CDH1* exon 3 sequence from the patient’s DNA with the deletion of G (Mut).

The electropherogram analysis revealed a heterozygous G base deletion in exon 3, introduced in a region of G base pairs repletion. The G deletion localized in position 360, in the precursor portion of E-cadherin, which resulted in a new reading frame and the generation of a stop codon 94 codons later in position 214 (p.His121Thrfs*94) (Figure 2). The same deletion was found in the proband and in some family members: his brother, a son, a niece, and a cousin (Figure 1).

The frameshift deletion in germline state is herein reported in a GC, while previously it has been described only in the somatic neoplastic cells (COSV55727175) of tumors from the esophagus and of the large intestine. The variant described in this manuscript was submitted to the Leiden Open Variant Database (LOVD, ID number 00276019) (https://databases.lovd.nl/shared/genes/CDH1) and reported in ClinVar SUB6906561 [8].

## 3. Discussion

This family study identified the c.360delG, p.His121Thrfs*94, a frameshift deletion localized in the precursor portion of the E-cadherin and causing the termination of the protein synthesis in four members of the six family members tested. A family history of cancer and germline variants are among the most important risk factors for cancer. The germline variants of several genes, including *CDH1, BRCA1*, *BRCA2*, and *APC*, are implicated in the onset of gastric cancer [9,10,11].

Reduced or absent E-cadherin expression, protein codified by the *CDH1* gene, is commonly associated with a poor prognosis in gastric cancer (GC) [12]. Indeed, the reduction of E-cadherin is positively associated with the mesenchymal–epithelium transition (MET), a biological process in which the epithelial cells can acquire motility and invasive capacities [13]. Besides, the reduction of E-cadherin promotes tumor cell proliferation by leading the accumulation of the β-catenin in the cytoplasm followed by its translocation into the nucleus and thus the activation of Wnt/b-catenin signaling [14]. A mechanism leading to a reduced E-cadherin expression is the presence of mutations in the *CDH1* gene resulting in the production of a non-functional protein or its loss, but other mechanisms e.g., epigenetic *CDH1* promoter hypermethylation, were also observed [12,15]. *CDH1* is a tumor-suppressor gene and its inactivation follows the classic hypothesis known as the “two-hit hypothesis” which requires that both *CDH1* alleles must be inactivated either by mutation or hypermethylation [16,17]. *CDH1* is a large size gene that shows a high number of variants, and the clinical significance of most of these *CDH1* variants is uncertain [18]. The clinicopathological significance of *CDH1* variants commonly necessitates clinical family history and in most cases also in vitro tests [18]. In individuals with the c.360delG frameshift deletion in heterozygosity, the “first-hit” is already present and thus they are more susceptible to developing gastric cancer [19]. The frameshift, probably by causing the loss of the E-cadherin, leads to reduced cell adhesion and a cellular transformation that favors tumor development in the familial clustering carriers [18,19].

Since the initial discovery of the pathogenetic *CDH1* mutations in three Maori families [20], more than 155 pathogenetic *CDH1* germline variants have been reported in gastric cancer, including numerous truncations, deletions, insertions, splice site variants, nonsense, silence, and missense variants [21]. The c.360delG frameshift deletion, located in exon 3, codifies for the precursor portion of the E-cadherin and causes the termination of the protein synthesis. The *CDH1* precursor region is necessary for the regulation of the E-cadherin production. Physiologically, this region controls the inactivation of the *CDH1* transcription to be an obstacle to the eventual overproduction of E-cadherin that could be detrimental for the morphogenesis of the cell. When E-cadherin production is necessary, the precursor region is cleaved or post-translationally modified [22]. The lack of the signal peptide, targeting the nascent protein into the endoplasmic reticulum during translation, might lead to aberrant intracellular trafficking with possible deleterious interference in cellular proteostasis. Moreover, the absence of E-cadherin at the cellular surface may aid the malignant transformation of cells explaining the different cancer manifestations within the HDGC syndrome. An association between the status of *CDH1* germline variants with the loss of the precursor region and clinical phenotypes of HDGC being more virulent has also been demonstrated [23]. Moreover, in families with truncating *CDH1* germline variants affecting the precursor region, an increased probability of colorectal cancer has been evidenced [23]. In *CDH1* germline mutation carriers, the possible explanation for the different intracellular transduction perturbations and variable cancer phenotypes might be the different levels of intracellular signal activation including different p-ERK, p-mTOR, and β-catenin levels or the differences in the truncating *CDH1* variants to subject *CDH1* mRNA transcripts to nonsense-mediated mRNA decay (NMD) [24,25]. A study reported that carriers with truncating *CDH1* variants escaping NMD are less affected by the early onset of gastric cancer [26]. Moreover, within the same family, the progression from indolent signet ring cell carcinoma (SRCC) foci to an aggressive HDGC phenotype was attributed to the activation of additional oncogenic events since it was found that in concomitance with the tumor development, the Ki-67 proliferation index and p53, pSrc (Tyr416) and pStat3 (Tyr705), the immunoreactivity detection increased [27]. Moreover, the aggressive phenotype is initially difficult to diagnose since the GC is often multifocal and located beneath an intact mucosal surface, observed from the microscopic lesions that emerge from the prophylactic total gastrectomies of HDGC patients.

In our proband’s family, four male members died from GC: the father, two uncles and his brother in a variable age range (Figure 1) and his mother who was diagnosed with breast cancer at the age of 50 years; no further clinical information was available for these patients. A *CDH1* genetic test carried out on the living relatives revealed a *CDH1* variant in three young carriers: the son, the niece, and the cousin of the proband (27, 34, 57 years, respectively) and in the proband (56 years old). The aunt of the proband, 93 years old, despite having the variant did not develop the disease. Furthermore, given her advanced age, a biopsy is not available to exclude the eventual presence of preclinical lesions. At the same time, other members of the proband’s family (an uncle and his brother) showed an invasive, aggressive HDGC and died at 31 and 59 years old, respectively. In *CDH1* mutation carriers, the reducing/prophylactic total gastrectomy (PTG) in early adulthood is the only current curative approach, considering that the gastroendoscopy often fails to detect early lesions [28]. The current consensus guidelines recommend PTG in early adulthood, generally between 20 and 30 years of age [1].

The proband and his son, after the detection of *CDH1* pathogenic frameshift deletion, immediately assent to the cancer surveillance Cambridge protocol and they did not exclude the possibility of proceeding with preventive gastrectomy [1]. Not all the proband’s family members have a GC, as other genetic or environmental factors may play a role in the penetrance and cancer risk. A better understanding of these factors in the future will improve the personalized risk assessment and clinical decision making for *CDH1* variant carriers.

## 4. Material and Method

Informed consent for genetic testing approval was obtained before the genetic testing. DNA was extracted from the peripheral blood and analyzed for the entire *CDH1* coding sequence, including the promoter region as previously published [29]. Briefly, 16 polymerase chain reaction (PCR) products were sequenced on an Applied Biosystems 3130 automated sequencer (Applied Biosystems, Foster City, CA, USA). The sequences were assembled with the CodonCode Aligner (CodonCode Corporation, MA, USA). The *CDH1* sequence variants identified were detailed using recommendations from the Human Genome Variation Society [30] and the interpretation of the sequence variants was based on the American College of Medical Genetics and Genomics guidelines [31].

## 5. Conclusions

Our report describes the family pedigree based on a germline truncating variant in the *CDH1* gene (c.360delG, p.His121Thrfs*94). We found the c.360delG variant encoding for a non-functional E-cadherin in the proband case, as well as in four family members among the seven cases tested. This study added clinical significance to a molecularly defined *CDH1* pathogenetic variant that occurs in a family at risk for GC. As this variant is found in the precursor region of the E-cadherin protein, the report highlights the role of this non-coding *CDH1* region in the pathogenesis of GC. Moreover, since the frameshift occurs in the precursor region, an inactive form of the E-cadherin, the results may have implications to better understand the clinical consequence of alteration involving the precursor gene region. Besides, it highlights the importance of accurate genetic counseling and the *CDH1* test analysis, although in patients aged > 50 years old.

In HDGC, *CDH1* variants are highly penetrant with carriers showing a lifetime risk of 70–80% to develop a GC, with at 80 years old a cumulative risk of 67–70% for men and 56–83% for women. Prophylactic gastrectomy is recommended in carriers of a pathogenic germline *CDH1* variant between 20 and 30 years [32].

## Abbreviations

HDGC	Hereditary diffuse gastric cancer
PCR	Polymerase chain reactions
MET	Mesenchymal–epithelium transition
NMD	Nonsense-mediated mRNA decay
PTG	Prophylactic total gastrectomy
